# Gait Difficulties and Postural Instability in Adrenoleukodystrophy

**DOI:** 10.3389/fneur.2021.684102

**Published:** 2021-06-17

**Authors:** Neha P. Godbole, Reza Sadjadi, Madeline A. DeBono, Natalie R. Grant, Daniel C. Kelly, Peter F. James, Christopher D. Stephen, M. David Balkwill, Richard F. Lewis, Florian S. Eichler

**Affiliations:** ^1^Department of Neurology, Massachusetts General Hospital, Boston, MA, United States; ^2^Harvard Medical School, Boston, MA, United States; ^3^Massachusetts Eye and Ear, Boston, MA, United States

**Keywords:** adrenoleukodystrophy, balance, myelopathy, spinal cord disease, gait, computerized dynamic posturography

## Abstract

**Background:** Gait and balance difficulties are among the most common clinical manifestations in adults with X-linked adrenoleukodystrophy, but little is known about the contributions of sensory loss, motor dysfunction, and postural control to gait dysfunction and fall risk.

**Objective:** To quantify gait and balance deficits in both males and females with adrenoleukodystrophy and evaluate how environmental perturbations (moving surfaces and visual surrounds) affect balance and fall risk.

**Methods:** We assessed sensory and motor contributions to gait and postural instability in 44 adult patients with adrenoleukodystrophy and 17 healthy controls using three different functional gait assessments (25 Foot Walk test, Timed Up and Go, and 6 Minute Walk test) and computerized dynamic posturography.

**Results:** The median Expanded Disability Status Scale score for the patient cohort was 3.0 (range 0.0–6.5). Both males and females with adrenoleukodystrophy showed impairments on all three functional gait assessments relative to controls (*P* < 0.001). Performance on walking tests and Expanded Disability Status Scale scores correlated with incidence of falls on computerized dynamic posturography, with the 25 Foot Walk being a moderately reliable predictor of fall risk (area under the ROC curve = 0.7675, *P* = 0.0038).

**Conclusion:** We demonstrate that gait difficulties and postural control deficits occur in patients with adrenoleukodystrophy, albeit at an older age in females. Postural deficits were aggravated by eyes closed and dynamic conditions that rely on vestibular input, revealing challenges to the interplay of motor, sensory and vestibular circuitry in adrenoleukodystrophy.

## Introduction

X-linked adrenoleukodystrophy (ALD) is a neurogenetic disorder of brain and spinal cord caused by mutations in the *ABCD1* gene, which result in decreased levels of ABCD1, a membrane protein that transports very long chain fatty acids into peroxisomes for degradation (1). In adulthood, ALD manifests as a non-inflammatory myelopathy referred to as adrenomyeloneuropathy (2–4), but patients remain at risk for developing brain inflammation characteristic of childhood ALD (5, 6). Brain inflammation is seen in males with cerebral ALD but rarely in female heterozygotes. While this most commonly leads to progressive neurological decline, active inflammation can also resolve and stabilize as self-arrested cerebral ALD (7). It is clear that myelopathy affects these males with stable brain lesions as well (8). Patients suffer from gait and balance difficulties across the phenotypic spectrum in adulthood (9–11), but little is known about the relative contributions of sensory loss, motor dysfunction, and postural control to gait dysfunction and fall risk. Multidimensional quantitative performance measures are needed to better characterize and define the association between sensory deficits and balance issues in both males and females with ALD.

Computerized dynamic posturography (CDP) assesses standing balance using a moveable force plate to simulate conditions that mirror those encountered in daily life (12). Using this platform, sensory organization testing (SOT) contains a set of six conditions to evaluate a patient's ability to integrate somatosensory, visual, and vestibular inputs to minimize sway (13). This tool has been used to assess postural instability in Parkinson disease (14) and to evaluate balance in older adults (15). CDP may therefore be an ideal tool to better characterize and quantitate the myelopathy of ALD. We sought to perform quantitative postural measures to assess standing balance, compare these measures with functional gait assessments, and identify potential predictors of fall risk in males and females with ALD.

## Materials and Methods

### Design

In this study, males and females over the age of 18 years with a diagnosis of ALD were recruited from the Leukodystrophy Clinic at Massachusetts General Hospital. All participants provided written informed consent and the study was approved by the local Institutional Review Board, the Mass General Brigham Human Research Committee. We included patients with genetically confirmed X-ALD, without radiographic evidence of active inflammatory disease on brain MRI (absence of lesional contrast enhancement). We excluded patients with the presence of other confounding neurological deficits that in the investigator's opinion could affect balance, being non-ambulatory (using a wheelchair), or an Expanded Disability Status Scale (EDSS) score of ≥ 7 determined by neurological examination (16). Neurological examinations were conducted by two neurologists with expertise in neuromuscular disorders (F.E. and R.S.). We also enrolled age-matched healthy control participants age ≥18 years.

### Retrospective Chart Review

We retrospectively reviewed medical charts of patients who attended the Massachusetts General Hospital Leukodystrophy Clinic from November 2017 to September 2019 and yielded 44 patients with ALD, of which three were excluded due to incomplete clinic notes. We extracted data on the presence of seven major neurological signs and symptoms including neuropathic pain, lower extremity sensory loss, gait disorder, use of a walking aid, balance difficulty, urinary urgency, fecal incontinence, and erectile dysfunction (in male patients). Balance difficulty was identified by either patient complaint and/or a positive Romberg sign documented in the clinic note during the neurological examination. These data were obtained in order to assess the extent of myelopathy-associated signs and symptoms with which our patient cohort presented.

We also recorded the presence of lesions as evident on brain MRIs performed as standard of care. The brain MRIs encompassed axial T2-weighted as well as pre- and post-contrast T1-weighted sequences. Non-inflammatory lesions were defined as lesions that did not show contrast enhancement on the post-contrast T1-weighted sequence. Active inflammatory lesions were defined as those displaying contrast enhancement. These patients with active inflammation were excluded from our study.

### Functional Gait Assessment

We assessed participants' gait disability using three functional outcome measures: the 25 Foot Walk (25FW) test, the Timed Up and Go (TUG) walking test, and the 6 Minute Walk (6MW) test. All walking tests were performed in a flat, unobstructed 30 m walkway. Start and finish lines for each of the tests were clearly marked and the average of two trials was recorded for both the 25FW and TUG. For the TUG, participants were instructed to rise from a chair without using the arm rests, walk 10 ft, pivot, and return to the chair again without using the arm rests for support. The time from when the participant arose from the chair and when they sat back down was recorded.

### Quantitative Balance Testing

We analyzed balance using the NeuroCom SMART Equitest posturography platform (13, 14) to assess postural sway, a proxy for standing balance. Standardized instructions were provided to all patients prior to testing. As a safety precaution, participants were strapped into a modified parachute harness prior to testing and a gait belt was used for additional support as necessary. Balance testing included the sensory organization testing (SOT), which measures the amount of sway as an equilibrium score from 0 to 100, with 100 being perfect stability and 0 representing a fall. Individuals without balance impairments can tolerate anterior/posterior sway of 12.5 degrees without falling. The equilibrium score is calculated by comparing the participant's anterior/posterior sway to the 12.5 degree maximum. Sway angles were computed based on the participant's center of gravity at an upright stance and during testing (17). The SOT captures both static and dynamic balance through six conditions in the following order:

1. Eyes open, stable surface, stable visual surround.
2. Eyes closed, stable surface, stable visual surround.
3. Eyes open, stable surface, sway-referenced visual surround.
4. Eyes open, sway-referenced surface, stable visual surround.
5. Eyes closed, sway-referenced surface, stable visual surround.
6. Eyes open, sway-referenced surface, sway referenced-visual surround.

The software reports individual scores for each condition and a composite equilibrium score based on the participant's performance across all six conditions. Three 20 s trials of each condition were conducted and best performance across the trials was used for analysis. We were particularly interested in assessing conditions that remove visual cues to unmask the proprioceptive deficits known to occur in patients with ALD. Falls during SOT were defined as losing balance and being caught by the safety harness, taking a step off of the platform, or using the walls of the machine for stability, similar to the falls criteria used in previous studies (14). To capture the potential for fall risk, the number of falls patients experienced during the SOT were compared to their performances on the functional gait assessments and EDSS score. Comparisons between groups were performed for each condition separately and for the composite equilibrium score. If a participant fell on all three trials of a testing condition, a maximum score could not be obtained, and they were excluded in the analysis involving that condition.

### Statistical Analysis

All statistical tests were performed in GraphPad Prism version 8.0.0 (San Diego, CA, USA) and significance was designated as two-tailed *P* < 0.05 with Bonferroni correction for multiple testing. Non-parametric testing with Bonferroni correction for multiple testing, including Mann-Whitney *U-*test, was used to compare the following groups: patients and healthy controls, male and female patients, patients who did not fall during SOT and patients who did fall during SOT.

Binormal receiver-operating characteristic (ROC) curves were generated for each of the gait assessments, (6MW, TUG, 25FW) by SOT falls. This was done to assess the capability of these measures to predict fall risk and determine cutoff values. Lastly, we used Spearman rank-order correlation to analyze the association between performance on clinical outcome measures and SOT scores as well as the number of falls during SOT.

## Results

### Clinical Description and Demographics

We enrolled 44 adult patients with ALD (22 male, 22 female) and 17 healthy control participants (nine male, eight female). Descriptive characteristics of 41 of 44 participants in the patient cohort are listed in Table 1. Thirteen of the 22 male patients had radiographic evidence of non-inflammatory white matter disease. The median age of the total cohort was 42 (range 19–77) years. Males were 37.5 (IQR 23–41.25) years and females were older at 52 (IQR 44.5–58.5) years (*P* < 0.001). The two most common symptoms experienced were lower extremity sensory disturbance and balance difficulty, both affecting 32 of 41 (78.0%) of patients. Of the 29 patients with a gait disorder, 11 (26.8% of total cohort) utilized a walking aid. Seven of 22 males (31.8%) reported erectile dysfunction. Based on a limited subset of patient data (*n* = 20) available from chart review, the median age of myelopathy onset was 30 (range 18–63) years. The median EDSS score for the total cohort was 3.0 (range 0.0–6.5). A score of 3 indicates a combination of moderate and mild disability in different functional systems while being fully ambulatory.

**Table 1:** Symptoms present on neurological examination and EDSS scores for the patient cohort stratified by age and sex.

**Age**	** <30 years**	**30–50 years**		**>50 years**	**Total**		
**Sex**	**Male (*n* = 7)**	**Male (*n* = 15)**	**Female (*n* = 9)**	**Female (*n* = 10)**	**Male (*n* = 22)**	**Female (*n* = 19)**	**All (*n* = 41)**
**Neurological symptoms**^a^							
Neuropathic pain	0 (0)	1 (6.7)	7 (77.8)	4 (40.0)	1 (4.55)	11 (57.9)	12 (29.3)
Lower extremity sensory disturbance	4 (57.1)	14 (93.3)	6 (66.7)	8 (80.0)	18 (81.8)	14 (73.6)	32 (78.0)
Gait disorder	1 (14.3)	13 (86.7)	8 (88.9)	7 (70.0)	14 (63.6)	15 (78.9)	29 (70.7)
Walking aid	0 (0)	6 (40.0)	2 (22.2)	3 (30.0)	6 (27.3)	5 (26.3)	11 (26.8)
Balance difficulty	3 (42.9)	13 (86.7)	8 (88.9)	8 (80.0)	16 (72.7)	16 (84.2)	32 (78.0)
Urinary urgency	0 (0)	6 (60.0)	6 (66.7)	7 (70.0)	6 (27.3)	13 (68.4)	19 (46.3)
Fecal incontinence	0 (0)	3 (20.0)	5 (55.6)	3 (30.0)	3 (13.6)	8 (42.1)	11 (26.8)
Erectile dysfunction	1 (14.3)	6 (40.0)	—	—	7 (31.8)	—	—
**EDSS Score**^b^	1.0 (0.0–3.0)	3.5 (0.0–6.0)	3.0 (0.0–6.0)	2.5 (1.5–6.5)	3.0 (0.0–6.0)	3.0 (0.0–6.5)	3.0 (0.0–6.5)

*EDSS, Expanded Disability Status Scale; n, number of participants.*

a *Symptoms are presented as absolute numbers (percentage).*

b *EDSS scores are presented as median (range)*.

### Functional Gait Assessments

All 44 participants enrolled completed the following three functional gait assessments: 6MW, TUG, 25FW. Compared to controls, patients with ALD had significantly shorter median 6MW distances [454 (IQR 348–515) m vs. 569 (IQR 513–628) m, *P* < 0.0001], and longer median times for TUG [8.33 (IQR 6.97–11.57) s vs. 5.78 (IQR 5.24–6.73) s, *P* < 0.0001] and 25FW [5.62 (IQR 4.83–6.74) s vs. 4.11 (IQR 3.77–4.84) s, *P* < 0.0001] (Table 2). Additionally, stratifying the patient cohort by sex showed no significant difference between males and females (6MW: *P* = 0.3483, TUG: *P* = 0.0686, 25FW: *P* = 0.1847).

**Table 2:** Performance on gait assessment outcomes for patients and controls.

**Outcome measure**	**ALD (*n* = 44)^a^**	**Control (*n* = 17)^**a**^**	**Test statistic^**b**^**	***P-*value**
6MW (m)	454 (348–515)	569 (513–628)	108	<0.0001^**^
TUG (s)	8.33 (6.97–11.57)	5.78 (5.24–6.73)	111.5	<0.0001^**^
25FW (s)	5.62 (4.83–6.74)	4.11 (3.77–4.84)	109	<0.0001^**^

*ALD, Adrenoleukodystrophy; n, number of participants; 6MW, 6 Minute Walk; TUG, Timed Up and Go; 25FW, 25 Foot Walk; IQR, Interquartile Range.*

a *Scores are reported as median (IQR).*

b *Differences between patient and control scores were assessed with Mann-Whitney U-tests.*

** *P < 0.01*.

### Quantitative Balance Testing

Results for CDP SOT by disease status are shown in Table 3. Significant differences were found between the patient and healthy control cohorts for the composite equilibrium score and all conditions (*P* < 0.01) except for condition four (eyes open, dynamic surface, stable visual surround). No significant differences were found between males and females (SOT overall *P* = 0.4595). Likewise, no significant differences were found between males with and without non-inflammatory lesions (SOT overall *P* = 0.9870). There was a weak negative relationship between SOT score and 25FW (*r* = −0.3797, *P* = 0.0110), and a moderate negative relationship between SOT and EDSS scores (*r* = −0.5574, *P* = < 0.001). Whereas, higher SOT scores represent better function, higher 25FW and EDSS scores represent more severe disability.

**Table 3:** Performance on SOT for patients and controls.

	***n***	**ALD (*n* = 44)^**a**^**	**Control (*n* = 17)^**a**^**	**Test statistic^**b**^**	***P-*value**
SOT condition 1	44	94 (92–96)	97 (96–97)	99	<0.0001^**^
SOT condition 2	43	90 (85–94)	95 (94–96)	118	<0.0001^**^
SOT condition 3	44	91 (86–94)	95 (93–96)	141	<0.0001^**^
SOT condition 4	44	85 (79–88)	87 (84–93)	248.5	0.0428
SOT condition 5	40	66 (56–72)	79 (74–82)	113.5	<0.0001^**^
SOT condition 6	38	69 (55–73)	80 (70–85)	111	<0.0001^**^
SOT equilibrium	44	67 (56–73)	81 (76–83)	84	<0.0001^**^

*ALD, Adrenoleukodystrophy; SOT, Sensory Organization Testing; n, number of participants; IQR, Interquartile Range.*

a *Scores are reported as median (IQR).*

b *Differences between patient and control scores were assessed with Mann-Whitney U-tests.*

** *P < 0.01*.

### Falls Risk Assessment

Falls data were compared in two ALD groups, with patients divided into categories of “no falls” or “falls” depending on what they experienced during the balance testing. Most SOT falls occurred during conditions five or six (91.9%). Of the 23 patients who fell during SOT, 21 fell only on conditions five or six (91.3%). Differences were found between the no falls and falls groups for all three walking assessments and EDSS score (Table 4). Compared to the no falls group, the falls group had a shorter median 6MW distance [366 (IQR 297–502) m vs. 476 (IQR 426–569) m, *P* = 0.0089], and longer median times for TUG [9.12 (IQR 7.57–13.02) s vs. 7.55 (IQR 6.55–8.56) s, *P* = 0.0126] and 25FW [6.51 (IQR 5.28–8.59) s vs. 4.94 (IQR 4.71–5.97) s, *P* = 0.0012]. The falls group also had higher EDSS scores [3.5 (IQR 2.5–4.0) vs. 2.0 (IQR 0.5–3.0), *P* = 0.0020]. ROC curves were generated for falls in each of the functional gait assessments. The 25FW test had the highest area under the curve (AUC) value, 0.7675 (IQR 0.6192–0.9158), while the TUG test had the lowest, 0.7100 (IQR 0.5459–0.8741). The cutoff values for each assessment were 373.4 m, 8.345 s, and 5.0 s for the 6MW, TUG, and 25FW test, respectively (Table 5). There were weak correlations between falls on SOT and clinical outcome measures, with the strongest relationships being between the 25FW and EDSS score (*r* = 0.4980, *P* = 0.0006 and *r* = 0.4976, *P* = 0.0006, respectively).

**Table 4:** Performance on gait assessments and EDSS scores for patients who did and did not fall on balance testing.

**Outcome measure**	**No falls (*n* = 21)^a^**	**Falls (*n* = 23)^**a**^**	**Test statistic^b^**	***P-*value**
6MW (m)	476 (426–569)	366 (197–502)	131.5	0.0089^**^
TUG (s)	7.55 (6.55–8.56)	9.12 (7.57–13.02)	136	0.0126^*^
25FW (s)	4.94 (4.71–5.97)	6.51 (5.28–8.59)	107	0.0012^**^
EDSS score	2.0 (0.5–3.0)	3.5 (2.5–4.0)	114	0.0020^**^

*n, number of participants; 6MW, 6 Minute Walk; TUG, Timed Up and Go; 25FW, 25 Foot Walk; IQR, Interquartile Range.*

a *Scores are reported as median (IQR).*

b *Differences between fallers and non-fallers scores were assessed with Mann-Whitney U-tests.*

* *P < 0.05.*

** *P < 0.01*.

**Table 5:** ROC analysis for predicting falls during SOT in patients with ALD.

**Outcome measure**	**Cutoff value**	**AUC (95% CI)**	**Sensitivity (%)**	**Specificity (%)**	***P-*value**
6MW (m)	373.4	0.7063 (0.5434–0.8691)	50	90	0.0256^*^
TUG (s)	8.345	0.7100 (0.5459–0.8741)	65	80	0.0231^*^
25FW (s)	5.0	0.7675 (0.6192–0.9158)	90	60	0.0038^**^

*ROC, Receiver Operator Characteristic; SOT, Sensory Organization Testing; ALD, Adrenoleukodystrophy; CI, Confidence Interval; AUC, Area Under Curve; 6MW, 6 Minute Walk; TUG, Timed Up and Go; 25FW, 25 Foot Walk.*

* *P < 0.05.*

** *P < 0.01*.

## Discussion

In this study, we used functional gait assessments and quantitative balance testing to demonstrate impairments of gait, balance, and posture in adults with ALD. Importantly, CDP revealed meaningful deficits in postural control in both males and females. These findings were associated with a higher incidence of falls during balance testing and did not correlate with presence of non-inflammatory brain lesions on MRI.

Our findings in females with ALD are congruent with prior studies and cannot be explained merely by the older median age of females compared with males in our cohort. A previous study of leg strength, tone, and vibration in patients with ALD found that sex differences were more pronounced in the earlier “mild” form of disease, but disease severity was comparable between males and females in the “moderate” and “severe” stages of disease (9). Our findings align with this study, suggesting that while females have later onset of symptoms, they may eventually suffer from a similar disease burden as males.

Functional gait assessments and CDP SOT in males and females with ALD revealed impairments on all three walking tests, as well as proprioceptive deficits demonstrated by removing visual cues during SOT. Walking distance and speed were comparable to prior studies (10). In our study, 13 male patients had evidence of non-inflammatory lesions on MRI at least 1 year prior to testing. These patients showed no significant differences on quantitative performance measures to patients without brain lesions, suggesting that long-term compensation may have occurred or that the myelopathy alone is a larger determinant of performance.

Importantly, adult patients with ALD struggled to maintain balance on conditions five and six of CDP testing, as indicated by the high number of falls. These conditions have a dynamic platform and eliminate or minimize the use of visual compensatory mechanisms, either by directly having the patient close their eyes or by changing visual perception through the use of a moving surrounding. The magnitude of change in these conditions was far greater than with moving surface alone without perturbation of the visual surrounds (condition four). Similar to other diseases with prominent dorsal column/proprioceptive involvement, our findings suggest that patients with ALD rely more heavily on their vision to maintain balance in dynamic conditions. A majority of falls occurred in conditions five and six, which is comparable to studies in Parkinson disease (18). These conditions challenge vestibular function (19), which could suggest that impairments in postural control may be aggravated by vestibular dysfunction and not visual processing alone. Future studies should include standard assessments of vestibular function and cognitive-visual processing, including yaw axis sinusoidal rotation and video head impulse tests.

Of note, the outcomes on all three walking tests and EDSS scores correlated with falling during the SOT. Literature in Parkinson disease has shown that slower times on the TUG walking test are associated with self-reported fall frequency (20). In pursuit of a more rigorous metric than anecdotal reporting, we determined the number of falls in real time through SOT. The ROC analysis showed that all three walking tests had moderate discriminatory ability, with AUC values >0.7. Of the three gait assessments, the 25FW test was the most sensitive predictor of falls during SOT. One reason for the higher discriminatory value of the 25FW in our study may lie in its ability to measure walking speed, which has been shown to be a good predictor of falls in older populations (21). The 6MW, which is a test of endurance, had the least discriminatory value of all three tests. This may indicate that patients with ALD are not falling due to fatigue. Unlike the much longer 6MW test, the 25FW test can be easily administered in the clinic setting. Poor performance on this measure may alert clinicians to a patient's need for orthotics or physical therapy, which has shown to improve walking and reduce falls in patients with neuromuscular disease (22).

There are a few limitations of this study. First, given that the majority of our patient population was seeking neurological evaluation at an academic medical center, our cohort may overrepresent symptom burden in the broader ALD population. Second, the older age of females in our cohort likely contributes to our findings that gait and balance difficulties show no significant differences between sexes. Additionally, our finding that most falls during SOT occurred in conditions five and six may partly be explained by participant fatigue. Other limitations of this study include the lack of inter-rater reliability validation and the relatively small sample size of participants. While there are limitations to our method of scoring falls on balance testing, such as the lack of standardization of what constitutes a fall on the force plate, it may provide clinically relevant information for determining fall risk. Future work should include wireless technology such as motion sensors to detect subtle changes in head and trunk movements and characterize specific gait impairments not addressed in the current study.

In conclusion, we demonstrate that visual perturbations aggravate balance measures in both males and females with ALD, beyond the impact of the myelopathy characterized by functional gait analysis. Dynamic posturography highlights that the most impacted conditions depend on visual and vestibular input. By using technology that identifies and integrates somatosensory, vestibular, and visual information, we address the intricate balance control system impacted in ALD. Importantly, performance on all walking assessments was associated with a higher incidence of falls documented during CDP testing. This appeared independent of non-inflammatory brain lesions on MRI, pointing to the need for improved measures of balance and vestibular function in clinical practice.

## Data Availability Statement

The raw data supporting the conclusions of this article will be made available by the authors, without undue reservation.

## Ethics Statement

This study involving human participants was reviewed and approved by Mass General Brigham Human Research Committee. The patients/participants provided their written informed consent to participate in this study.

## Author Contributions

FE conceptualized and designed the study. NPG and RS performed the statistical analysis. NPG, RS, FE, CS, MB, and RL interpreted the data. NPG, MD, NRG, DK, PJ, and MB contributed substantially to the acquisition of data. NPG wrote the first draft of the manuscript. RS, MD, NRG, CS, MB, RL, and FE critically revised the manuscript for important intellectual content. All authors contributed to manuscript revision, read, and approved the submitted version.

## Conflict of Interest

FE reports sponsored research contracts with Minoryx Therapeutics and consultancies with Autobahn Therapeutics, Minoryx Therapeutics, and SwanBio Therapeutics. The remaining authors declare that the research was conducted in the absence of any commercial or financial relationships that could be construed as a potential conflict of interest.

## Abbreviations

ALD: X-linked Adrenoleukodystrophy
AUC: Area Under the Curve
CDP: Computerized Dynamic Posturography
EDSS: Expanded Disability Status Scale
ROC: Receiver-Operating Characteristic
SOT: Sensory Organization Testing
25FW: 25 Foot Walk
TUG: Timed Up and Go
6MW: 6 Minute Walk.
